# Total Elbow Arthroplasty

**DOI:** 10.2174/1874325001105010115

**Published:** 2011-03-16

**Authors:** Joaquin Sanchez-Sotelo

**Affiliations:** Department of Orthopedic Surgery, Mayo Clinic, Rochester, MN 55905, USA

**Keywords:** Arthroplasty, elbow, rheumatoid arthritis, elbow fractures, osteoarthritis.

## Abstract

Total elbow arthroplasty has continued to evolve over time. Elbow implants may be linked or unlinked. Unlinked implants are attractive for patients with relatively well preserved bone stock and ligaments, but many favor linked implants, since they prevent instability and allow replacement for a wider spectrum of indications. Inflammatory arthropathies such as rheumatoid arthritis represent the classic indication for elbow arthroplasty. Indications have been expanded to include posttraumatic osteoarthritis, acute distal humerus fractures, distal humerus nonunions and reconstruction after tumor resection. Elbow arthroplasty is very successful in terms of pain relief, motion and function. However, its complication rate remains higher than arthroplasty of other joints. The overall success rate is best for patients with inflammatory arthritis and elderly patients with acute distal humerus fractures, worse for patients with posttraumatic osteoarthritis. The most common complications of elbow arthroplasty include infection, loosening, wear, triceps weakness and ulnar neuropathy. When revision surgery becomes necessary, bone augmentation techniques provide a reasonable outcome.

Replacement arthroplasty of the elbow is in constant evolution. Although it was initially used mainly in patients with inflammatory arthritis, its indications were expanded to other conditions, which place higher demands on the implants and seem to lead to higher failure rates [1]. Elbow arthroplasty presents some unique peculiarities. Compared to the hip and knee joints, the elbow is relatively small and its stability depends greatly on ligamentous integrity. Linked semiconstrained elbow arthroplasties became popular in the United States and central Europe; these inherently stable implants raise the concern of increased contact pressures on the already thin polyethylene. Unlinked arthroplasties, popular in the United Kingdom and Asia, may have better tribological properties but are at risk for instability and decreased elbow extension.

Elbow arthroplasty is further complicated by the need to violate the extensor mechanism for exposure, the increased risk of infection, the role of the radial head, and potential clinical problems related to the ulnar nerve. Present and future innovations may include the use of linkable implants, alternative bearing surfaces, uncemented fixation, distal humerus hemiarthroplasties, unicompartimental arthroplasties, implantation with the aid of computerized navigation systems, and improved revision systems.

## 1 MATERIALS AND DESIGNS

### 1.1 Implant Types

There is some confusion regarding the types of implants available to replace the elbow joint. In general, there are two broad categories of implants, which differ in the presence or absence of a mechanism linking the humeral and ulnar components (Table **1**). A common misconception is to equate linking to constraint: some unlinked implants are more constrained than their linked counterparts.

#### Linked/Coupled Implants

The distinguishing feature of this category of implant is the physical linking of the humeral and ulnar components at the time of surgery in order to avoid subluxation or dislocation episodes. Early linked implants were constrained hinges that only allowed flexion and extension. These implants were associated with a high failure rate secondary to the transmission of high stresses to the implant-cement-bone interface and other design flaws. Currently, most linked implants are semiconstrained: their linking mechanism behaves as a sloppy hinge, allowing some rotational and varus-valgus play. Semiconstrained implants are believed to transmit less stress to the implant interfaces, which associated with other design improvements have resulted in more reliable long-term fixation.

**Fig. (1):**
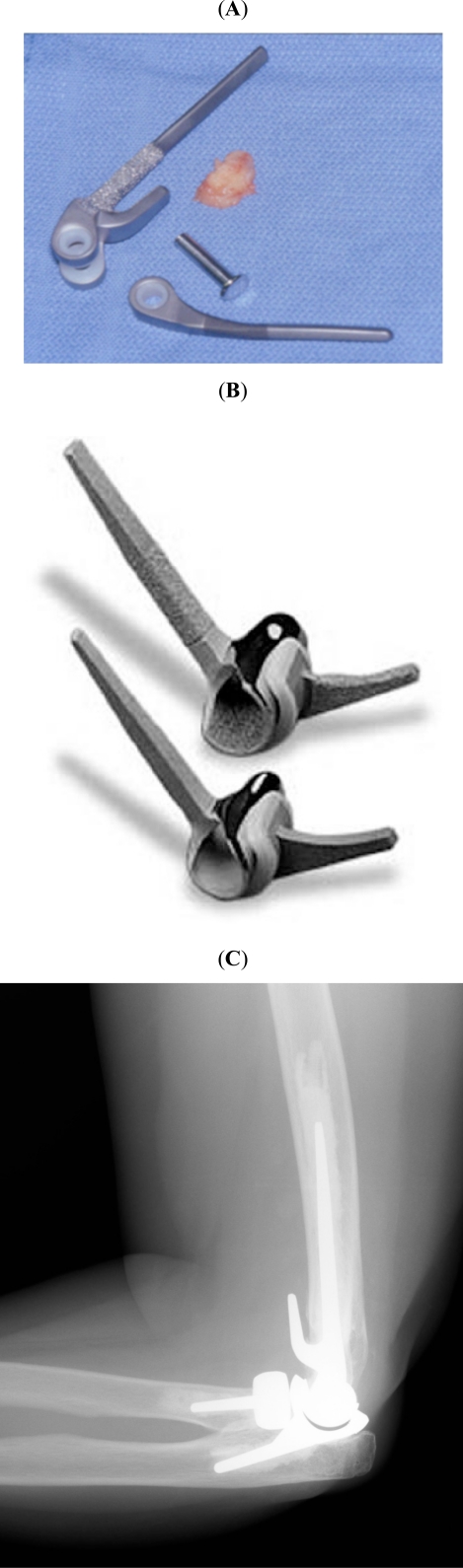
Some examples of implants used to replace the elbow joint: **(A)** Coonrad-Morrey linked semiconstrained elbow arthroplasty, **(B)** Kudo unlinked minimally constrained elbow arthroplasty, **(C)** Latitude anatomic linkable prosthesis.

The linked semiconstrained implant most commonly used currently is the Coonrad-Morrey prosthesis (Fig. **1A**). The humeral component is porous-coated distally and presents an anterior flange, which increases the rotational stability of the implant and neutralizes the extension forces transmitted to the implant interface. The benefit of an anterior flange has been investigated for other implants as well [2]. The ulnar component has a plasma-spray metallic coating in its proximal third. Both components are intended to be fixed with polymethylmethacrilate. The components are linked with a cobalt-chrome axis pin, which articulates with the polyethylene bushings of the ulnar and humeral components and allows approximately 10 degrees of varus-valgus and rotational laxity. Other linked implants are enumerated in Table **1**.

#### Unlinked/Uncoupled Implants

In this kind of arthroplasty the components are not mechanically linked. Maintenance of prosthesis congruency depends on the adequate position of each component, ligamentous integrity, and the dynamic stabilizing effect of the musculature. Most of these implants provide a more or less anatomic resurfacing of the distal humerus and proximal ulna; some incorporate a radial head component. The most popular unlinked implants are the Souter-Strathclyde and the Kudo prostheses (Fig. **1B**). Other unlinked implants are listed in Table **1**.

### 1.2 Advantages and Disadvantages of the Different Kinds of Implants

The clinical outcome and long-term survivorship differs from implant to implant, and the results obtained with a given linked or unlinked implant cannot be extrapolated to other members of the same implant family. However, there are a few advantages and disadvantages of each of these two design philosophies (Table **2**).

Linked implants ensure joint stability, even in the presence of severe bone loss or ligamentous insufficiency. These implants not only eliminate one of the main complications of unlinked implants, namely dislocation, but also allow a more aggressive soft-tissue release in patients with preoperative stiffness and deformity, which allows more reliable restoration of elbow motion. On the other hand, the increased constrained associated with implant linking results in increased tension on both the joint surface and the interfaces, which may facilitate polyethylene wear and component loosening. Semiconstrained implants did represent an improvement, but well-fixed semiconstrained implants are at risk for accelerated wear in the presence of ligamentous imbalance.

Some linked implants also allow replacement in the presence of severe bone loss. Many unlinked designs require the humeral condyles and ulnar notch for component fixation. Bone loss compromises fixation of this kind of components and may render the medial or lateral ligament complexes insufficient if the epicondyles are affected. In addition, patients with severe preoperative stiffness may require non-anatomic implantation of the humeral component to raise the joint line, which makes the use of unlinked implants more complicated.

However, linked implants do have substantial disadvantages, especially when they are constrained. In those situations where the remaining bone stock and ligamentous structures are adequate, unlinked implants are at least theoretically at less risk of mechanical failure secondary to wear, osteolysis and loosening. As a general rule, the stems of unlinked implants are shorter; this is especially beneficial when revision or resection is required. Some anatomic unlinked humeral components may also be used as hemiarthroplasties.

The need for a radial head implant is controversial. On one side, patients with an arthritic radial head or a previous radial head resection may benefit from the use of a radial implant, which may increase stability and result in a greater improvement on the lateral side of the joint. However, from a technical point of view it is difficult to achieve proper alignment and tracking of the radial head implant, and this component is potentially one more source or wear, osteolysis and loosening.

Currently published data seem to favour the use of linked semiconstrained implants. Little *et al*. [3] recently published a systematic review of the literature on elbow arthroplasty. The overall revision rate has been similar for linked and unlinked implants (11 *vs* 13 per cent). However, radiographic loosening seems to be higher with unlinked implants (especially the humeral component of the Souter prosthesis). The functional results are similar with the exception of elbow extension, which seems to be better with linked implants. On a separate study, Levy *et al. *reported a higher rate of revision for unlinked compared to linked implants [4].

### 1.3 Modern Implants

Recently designed implants have maintained some of the classic features recognized to improve the outcome of elbow arthroplasty (such as the use of a flange), but provide three potential advantages:

- The bearing surface design allows the use of a thicker polyethylene subjected to less contact pressure.
- The instrumentation and design allow a more anatomic reconstruction with more attention being paid to reproduction of the anatomic center of rotation.
- The components may be linked after being completely seated.

The Latitude system probably is the best example of this new generation of elbow arthroplasty (Fig. **1C**). This modular system is linkable, meaning that the surgeon may choose at the end of the case to leave the implant linked or unlinked depending on his intraoperative assessment of stability. In addition, this system allows conversion of a distal humerus hemiarthroplasty to a total elbow arthroplasty without revising the humeral stem.

## 2 INDICATIONS AND CONTRAINDICATIONS

Inflammatory arthropathies, such as rheumatoid arthritis, represent the classic indication of elbow arthroplasty. Those patients with more severe involvement (Mayo Clinic stage III to V) experience great improvements in pain and function. In addition, the polyarticular nature of these conditions may limit the overall activity level of these patients, with a low rate of wear and loosening. In the earlier stages of rheumatoid arthritis, there is usually enough bone stock and ligamentous integrity to allow the use of unlinked implants.

The successful outcome of elbow arthroplasty in inflammatory conditions prompted its use for the treatment of other conditions (Table **3**). Posttraumatic elbow osteoarthritis represents one of the most difficult conditions to treat. Some patients may improve with alternative surgical procedures, such as interposition arthroplasty, but pain relief is not completely reproducible and some patients may experience postoperative instability. Elbow arthroplasty provides a more reliable outcome, but these younger, more active patients are at risk for early mechanical failure [1]. In general, elbow arthroplasty is best avoided in patients under the age of sixty.

Acute comminuted distal humerus fractures in elderly patients or those with previous articular degeneration has emerged as one of the most common indications for elbow arthroplasty in some countries [5]. Stable internal fixation is difficult to obtain in these circumstances, and arthroplasty is used successfully for other fractures (femoral neck, proximal humerus). It is important to emphasize that this is a selective indication, as most patients with distal humerus fractures are best treated with open reduction and internal fixation.

Other indications for elbow arthroplasty include the salvage of distal humerus nonunion in elderly patients, large posttraumatic defects, as well as elbow reconstruction after tumor resection. Primary osteoarthritis of the elbow usually affects younger patients and is treated successfully in many patients with joint debridement procedures such as osteocapsular arthroplasty.

## 3 SURGICAL TECHNIQUE OVERVIEW

### 3.1 Surgical Exposure

Most of the surgical approaches used for implantation of an elbow arthroplasty require mobilization of the elbow extensor mechanism. Subcutaneous ulnar nerve transposition is routinely performed by most surgeons. The author’s preferred exposure is the triceps-reflecting Bryan-Morrey approach [6]; other surgeons prefer to split the triceps or use an extended lateral-sided Köcher approach. Triceps-preserving approaches are desirable whenever possible [7].

The approach described by Bryan and Morrey involves detaching the triceps off the olecranon reflecting it from medial to lateral maintaining its continuity with the anconeus and the forearm fascia. This approach provides ample exposure of the joint and allows a secure reconstruction of the extensor mechanism, although it is associated with some risk of lateral subluxation of the triceps and weakness in extension.

Splitting the triceps in the midline with detachment of its medial and lateral halves from the olecranon also provides a good exposure. The main advantage of this approach is maintenance of the extensor mechanism centralized over the olecranon, but transmuscular approaches are in general less appealing and the repair of the medial half is some times unsatisfactory.

In some specific circumstances, it is possible to perform the replacement by working on both sides of the triceps [7, 8]. This approach is mostly indicated in the presence of a substantial bone defect at the distal humerus (secondary to trauma or tumor resection), as well as in acute distal humerus fractures and nonunion of the distal humerus, where the distal fragments are resected.

### 3.2 Bony Preparation and Component Insertion

The bony preparation is different for each particular system. Most components are stemmed and require preparation of the humeral and ulnar canals with rasps and broaches. The author uses the Coonrad-Morrey system. With this system, the humeral side is prepared first after exposing the joint and releasing the lateral and medial collateral ligaments. The humeral canal is identified and used as a reference to cut a yoke-shaped segment of the distal humerus to accommodate the distal part of the humeral component. Next, the canal is prepared to accept the stem and the anterior cortex of the distal humerus is exposed for future contact with a bone graft placed behind the anterior flange of the humeral component. The ulnar canal is opened at the mid-portion of the trochlear notch and the canal prepared with right or left broaches. The components are then cemented in place with antibiotic-loaded polymethylmethacrylate placing a bone graft between the anterior humeral cortex and the humeral flange. The components are then linked together.

The ulnar canal is usually relatively narrow, which requires the use of small flexible cannula to introduce the cement, which should be applied very early. Preoperative stiffness or deformity usually requires extensive soft-tissue balancing and releases. Limited extension may be corrected by anterior capsular release and proximal placement of the humeral component with elevation of the joint line. Limited flexion is corrected by posterior capsular release and occasionally resection of the anterior aspect of the coronoid.

### 3.3 Postoperative Management

The goal of the early phase of postoperative treatment consists in limited postoperative edema. The elbow is immobilized in extension with an anterior plaster splint and a bulky dressing and the upper extremity is kept elevated. When a linked arthroplasty is used, elbow motion without protection may be initiated in the first few days after surgery depending on the aspect of the wound and the quality of the extensor mechanism reconstruction. Most surgeons keep the elbow immobilized for approximately two weeks after using an unlinked elbow arthroplasty to protect the ligamentous structures and decrease the risk of instability. A nocturnal extension splint is useful for the first few weeks after surgery when there is a marked preoperative flexion contracture. Elbow extension against resistance should be avoided whenever the extensor mechanism has been violated for exposure.

Polyethylene wear is the main limiting factor for the survivorship of current elbow designs. Prior to surgery patients should understand the need to protect their upper extremity. Empirically, patients are recommended to avoid lifting with the involved upper extremity more than 2 pounds on a repetitive basis or more than 10 pounds on a single event.

## 4 CLINICAL RESULTS

### 4.1 Chronic Inflammatory Arthritis

**Fig. (2):**
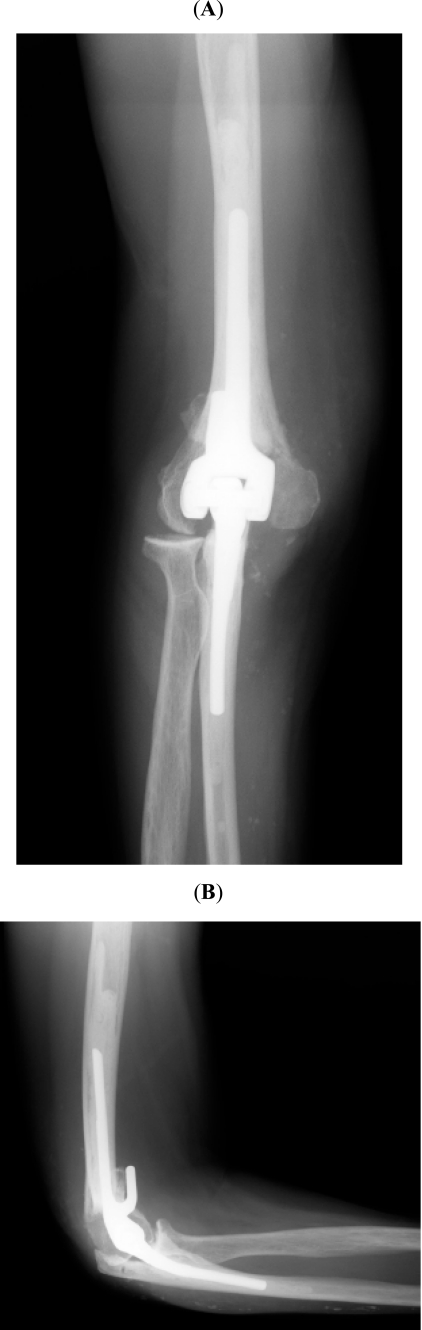
Anteroposterior **(A)** and lateral **(B)** radiographs after elbow arthroplasty for rheumatoid arthritis.

Several studies have documented the outcome of elbow arthroplasty in rheumatoid arthritis using both linked and unlinked implants. Gill and Morrey [9] published the results obtained in 78 consecutive rheumatoid elbows using the Coonrad-Morrey design. At most recent follow-up, 97 per cent of the patients had no or mild pain and the mean arc of motion was from 28 degrees of extension to 131 degrees of flexion. The main complications of this series included deep infection (2 cases), aseptic loosening (2 cases), triceps avulsion (3 cases), periprosthetic fractures (2 cases), and ulnar component fracture (1 case). Survivorship free of revision was 92.4% at ten years (Fig. **2**). Gschwend *et al*. [10] published the results using the GSB III prosthesis in 65 elbows, 32 of which were rheumatoid, followed for a minimum of 10 years. Overall clinical results were satisfactory and the main complications included infection (6 per cent), loosening (4.6 per cent) and component disengagement (13.6 per cent).

Van der Lugt *et al*. [11] published the results obtained in 204 rheumatoid elbows replaced using the Souter-Stractclyde prosthesis and followed for a mean of 6.4 years. At most recent follow-up, only 6 patients complained of pain at rest. Complications included infection (10 cases), humeral loosening (22 cases), and dislocation (4 cases). Kudo *et al*. [12] published the results obtained in 43 elbows replaced with the Kudo prosthesis and followed for a mean of three years; good or excellent results were obtained in approximately 86% of the patients, although some experienced loss of extension. Willems and De Smet published the results of 36 Kudo prosthesis in rheumatoid elbows; the main reported complications included infection (1 case), instability (2 cases), and loosening (6 cases) [13].

We recently reviewed the Mayo Clinic experience using a linked semiconstrained elbow arthroplasty in rheumatoid arthritis. 461 consecutive Coonrad-Morrey arthroplasties were followed for a mean of 8 (range, 2 to 25) years. At most recent follow-up, 418 implants (90.7%) had not been revised, 10 (2.2%) had been removed or revised for infection, 25 (5.4%) had been revised for loosening, 8 elbows (1.7%) had been revised for polyethylene wear, and 3 patients underwent internal fixation of a periprosthetic fracture. Seventeen additional elbows required debridement for deep infection (overall infection rate, 5.8%). Revision for polyethylene wear was performed between 10 and 17 years after surgery in all but one of the eight elbows. Twenty-year survivorships were 90% (95CI 79-94%) free of revision for loosening, 78% (95CI 65-89%) free of revision for mechanical failure, and 72% (95CI 58-85) free of revision for mechanical failure or deep infection.

In general, most patients with rheumatoid arthritis experience satisfactory pain relief and functional improvement with both linked and unlinked implants. Most patients also maintain a good arc of motion and the rate of mechanical failure is small. Some authors believe that the outcome of elbow arthroplasty is similar to the outcome of hip and knee arthroplasty in rheumatoid patients [9, 10]. Linked arthroplasties allow the treatment of a wider spectrum of pathology, including patients with more extensive involvement, bone defects and instability.

### 4.2 Trauma

#### Posttraumatic Osteoarthritis

This is one of the most common conditions affecting the elbow joint. Postoperative pain and stiffness are common sequels of elbow trauma. The first step in the evaluation of these patients is to determine how much the articular surface contributes to the patient’s symptoms. Patients with a symptomatic articular surface experience pain with resisted flexion and extension in the mid-arc of motion. The status of the articular surface may be evaluated with radiographs and CT scan.

When the articular surface is responsible for most symptoms, the alternative surgical options are somewhat limited and not totally satisfactory. Arthroscopic debridement is more reliable for impingement pain. Interposition arthroplasty includes placement of a layer of cutis, fascia lata or Achilles tendon allograft interposed between the humerus and ulna and temporary distraction of the joint with an articulated external fixator for approximately 6 weeks. This procedure is more reliable for restoration of motion than pain relief [14, 15]. Other procedures, such as osteoarticular allografts or elbow fusion, have a high rate of complications [16] or are poorly accepted by patients [17]. Elbow arthroplasty is very attractive as it provides the best early functional results; however, it is associated with a worrisome rate of mechanical failure especially in younger patients [18].

Schneeberger *et al*. [18] published the results of a study of 41 patients with posttraumatic osteoarthritis using the Coonrad-Morrey prosthesis. The mean age of the patients at the time of surgery was 57 years (range, 32 to 82 years) and the mean follow-up time was five years. Seventy-three per cent of the patients had no or mild pain and the results were considered satisfactory in 83 per cent of the cases. However, there was a 27 per cent complication rate, including five ulnar component fractures and two revisions for polyethylene wear. These authors concluded that elbow arthroplasty should be relatively contraindicated in patients planning to perform substantial physical activities with the involved upper extremity or are not able to comply with the previously mentioned postoperative restrictions.

We recently updated the Mayo Clinic experience with total elbow arthroplasty in post-traumatic osteoarthritis. Eighty-five consecutive patients underwent semiconstrained TEA for post-traumatic arthritis. Sixty-nine elbows with a retained primary prosthesis were followed for an average of 9.1 years (range 2-20.5). Sixteen primary arthroplasties (19%) failed secondary to isolated bushing wear (7), infection (4), component fracture (3), or component loosening (2). Four additional arthroplasties showed radiographic signs of loosening and three had substantial radiographic wear. Total elbow arthroplasty was associated with statistically significant gains in pain relief, motion, and MEPS scores (p<0.002). Forty seven (68%) patients achieved good or excellent clinical results using objective criteria and 74% were subjectively satisfied with their outcomes at final follow-up. Kaplan Meier analysis demonstrated a 15-year survivorship of 70% for revision or resection for any reason, 73.7% for revision for mechanical failure, and 90.2% for aseptic loosening.

The relatively high mechanical failure rate of elbow arthroplasty in patients with posttraumatic osteoarthritis has been the main driving force for the development of newer implants with supposedly better wear patterns. There are no published studies on the outcome of these new designs. An alternative strategy in younger patients is to offer them an interposition arthroplasty as their first procedure as long as they understand that pain relief is not reliable; fortunately, the outcome of replacement after failed interposition arthroplasty is equivalent to that of patients without previous interposition [19].

#### Distal Humerus Fractures

Open reduction and internal fixation is the treatment of choice for most distal humerus fractures. However, the outcome of internal fixation may be compromised in a selective group of patients with extensive comminution, osteopenia or previous articular pathology. For elderly patients in this situation, elbow arthroplasty probably represents a better alternative.

There are different philosophies for the use of elbow arthroplasty in distal humerus fractures. The author’s preferred strategy is to work through a bilaterotricipital approach, resect the fractured fragments, and complete the arthroplasty. When the distal fragments are resected, the collateral ligament complexes and the flexor-pronator and extensor-supinator groups are detached. A linked arthroplasty is needed to compensate for the ligamentous insufficiency. The forearm muscular groups are sutured to the triceps to seal the joint; interestingly, resection of the humeral condyles does not seem to affect grip strength or strength in flexion, extension, pronation or supination [20]. Other philosophy consists in fixing the condyles to preserve the integrity of the collateral ligaments and replace the articular surface with a distal humerus hemiarthroplasty or a total elbow replacement.

**Fig. (3):**
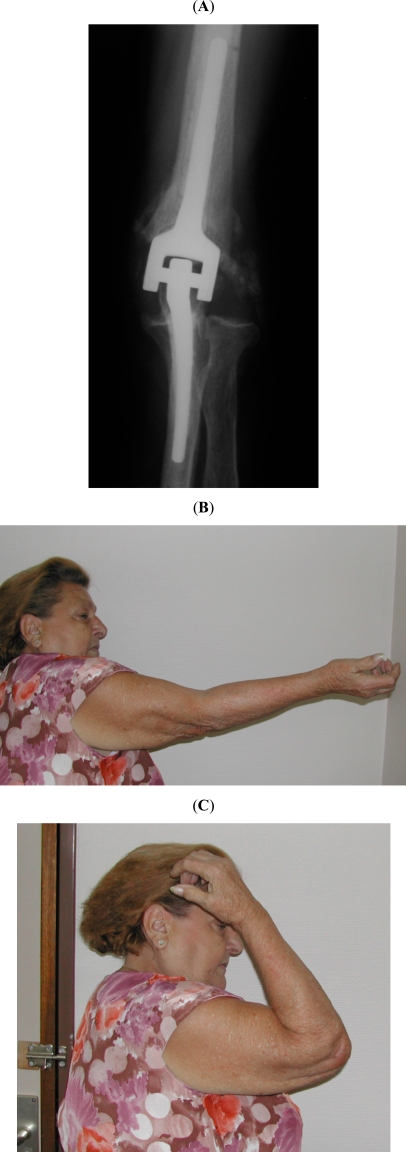
Postoperative radiograph **(A)** and final range of motion (**B** and **C**) after elbow arthroplasty for an acute distal humerus fracture in an elderly female patient.

The outcomes of total elbow arthroplasty in selected patients with complex distal humerus fractures are quite satisfactory. Kamineni and Morrey recently reviewed the results obtained in a consecutive series of 43 patients followed for a mean of seven years [5]. Most patients achieved a satisfactory Mayo Elbow Performance Score and the mean arc of motion was from 24 degrees of extension to 131 degrees of flexion (Fig. **3**). However, nine patients required a reoperation, including 5 cases of component revision. Other authors have published similar outcomes [21-24]. Frankle *et al*. [21] performed an interesting comparative study between internal fixation and arthroplasty in 24 fractures affecting women over 65 years old and obtained a better in the arthroplasty group.

#### Distal Humerus Nonunion

The salvage of distal humerus nonunion in selected patients represents a good indication for elbow arthroplasty. Most distal humerus nonunions are treated with internal fixation and bone grafting. However, elderly patients with osteopenia and very limited bone stock may be benefit more from elbow arthroplasty. Morrey and Adams published the results obtained in 36 patients with a mean age of 68 years followed for an average time of 4 years after elbow arthroplasty for distal humerus non-union [25]. Results were rated as satisfactory in 86 per cent of the cases; there were two infections and three patients with excessive polyethylene wear. We recently updated the Mayo Clinic experience using elbow arthroplasty for the salvage of 92 distal humerus nonunion. At a mean follow-up of 6.7 years (range, 2 to 20 years), 79 per cent of the patients had no or mild pain and mean range of motion was from 22 degrees of extension to 135 degrees of flexion. Complications included aseptic loosening in 16 patients, component fracture in 5 patients, deep infection in 5 patients and bushing wear in one patient.

### 4.3 Other Indications

Total elbow arthroplasty has also been successfully used in patients with severe stiffness or ankylosis [26], gross instability secondary to large bony defects [27], haemophilic arthropathy [28], and reconstruction after tumor resection [29].

## 5 COMPLICATIONS

### 5.1 Infection

Deep periprosthetic infection affects the elbow more commonly than other joints. This is attributed to the thin soft-tissue envelope of the elbow as well as the higher risk of infection in patients with relative immune suppression secondary to inflammatory conditions or failed previous surgical procedures for trauma. Currently, the incidence of infection after elbow arthroplasty is estimated to be between 2 and 4 per cent [3, 30] Antibiotic-loaded polymethylmethacrylate is used routinely for implant fixation in an effort to decrease the rate of infection. Acute infections may be treated with irrigation, debridement, polyethylene exchange and retention of the components. Chronic infections may be treated with two-stage reimplantation or resection depending on the nature of the infection, patient needs and remaining bone and soft-tissues.

### 5.2 Ulnar Neuropathy

The overall rate of ulnar neuropathy is difficult to estimate as patients with sensory symptoms are not reported accurately on most published studies about elbow arthroplasty. The incidence of severe ulnar neuropathy probably is around 5 per cent [3]. Most surgeons recommend routine subcutaneous ulnar nerve transposition at the time of arthroplasty to prevent postoperative ulnar nerve dysfunction.

### 5.3 The Extensor Mechanism

The rate of extensor mechanism dysfunction is also difficult to analyze in the published literature and probably is underestimated. In *Little et al*.’s systematic review of the literature the incidence of triceps insufficiency was 3 per cent [3]. Poor soft-tissue quality as present in many patients with rheumatoid arthritis may affect the quality of the triceps repair at the end of surgery. Patients with symptomatic dysfunction of the extensor mechanism may benefit from surgical reconstruction of the extensor mechanism using either an anconeus rotation flap or an Achilles tendon allograft [31].

### 5.4 Instability

Unlinked elbow arthroplasty may be complicated by subluxation or dislocation. The rate of dislocation is approximately 5 per cent; the overall rate of instability (dislocation or subluxation) is about 15 per cent [3]. There are different treatment options. Dislocation presenting in the first few weeks after surgery may respond to closed reduction and immobilisation. However, most patients with instability require revision surgery for ligamentous reconstruction or revision to a linked elbow arthroplasty [32].

### 5.5 Mechanical Failure

The overall rate of aseptic loosening after elbow arthroplasty probably ranges between 5 and 10 per cent, and it is different for different implant designs. According to *Little et al.*, the published aseptic loosening rate is 2 per cent for the Coonrad-Morrey prosthesis, 8 per cent for the Souter prosthesis, and 18 per cent for the Kudo prosthesis [3]. Polyethylene wear and osteolysis, component fracture, and component disengagement are additional modes of mechanical failure whose rate is difficult to estimate. Polyethylene wear probably is the limiting factor for the durability of elbow arthroplasty in young active patients.

### 5.6 Periprosthetic Fractures

Elbow periprosthetic fractures are classified based on the location of the fracture, the fixation of the components and the need to use special reconstructive techniques for bone loss [33]. Most fractures of the humeral condyles may be treated nonoperatively provided they are not associated with instability in the case of unlinked prosthesis. Most periprosthetic fractures require component revision and internal fixation using plates or cortical strut allografts [33, 34].

## 6 REVISION SURGERY

The increasing use of elbow arthroplasty, especially in younger patients with increased functional demands, has resulted in a substantial increase in the prevalence of revision surgery. Most revisions surgeries require the use of a linked prosthesis, as the severity of bone loss and ligamentous insufficiency in the revision setting rarely permits the use of an unlinked implant.

A careful preoperative evaluation of the patient prior to revision surgery is critical for success. The physical examination should consider the condition of the skin, location of previous incisions, range of motion, joint stability, muscle function and strength, as well as the location and function of the ulnar nerve. The possibility of infection should always be considered and investigated with baseline laboratory studies including white cell count, sedimentation rate and C reactive protein. Joint aspiration for cell count and cultures should probably be considered in every patient and is mandatory if there is a high suspicion of infection or the parameters mentioned above are elevated. Preoperative radiographs should also be analyzed carefully to evaluate the fixation of the components and the severity of bone loss.

A few basic principles apply to all revision cases. The skin overlying the elbow joint is very fragile; the previous skin incision should be used whenever possible and the soft tissues should be handled with extreme care. The ulnar nerve should be identified and protected in all cases; complex humeral reconstructions also require identification and protection of the radial nerve. In many instances, component revision may be performed working on both sides of the triceps, especially in the presence of severe bone loss. Component and cement removal should be done with extreme care, as intraoperative perforations and fractures can occur easily; the use of high-speed burs and flexible cannulated canal reamers is recommended, and sometimes it is necessary to create a controlled osteotomy of the humerus or ulna. In the absence of infection, it is reasonable to preserve well-fixed cement and use cement within cement technique for implant fixation.

King *et al*. reported the initial Mayo Clinic experience in a consecutive series of 41 revision elbow arthroplasties followed for a mean of 6 years [35]. Most patients experience a substantial improvement in pain and function and many were able to resume activities of daily living. However, there was a high incidence of complications, including intraoperative fractures and radial or ulnar nerve dysfunction. More recent studies have documented a high success rate with revision techniques used in the presence of bone loss, including cortical strut allografts, impaction grafting and allograft-prosthetic composites [33, 34, 36, 37]. In general terms, allograft-prosthetic composites have provided inferior results compared to other techniques.

## 7 SUMMARY

The field of elbow arthroplasty continues to experience substantial improvements. Currently, elbow replacement represents a successful treatment alternative for patients with inflammatory conditions as well as selected patients with posttraumatic osteoarthritis, elderly patients with low, comminuted distal humerus fractures, the salvage of distal humerus nonunion, ankylosis, haemophilic arthropathy, and elbow reconstruction after tumor resection. Some linked arthroplasty designs seem to be associated with a better outcome and allow the management of a wider range of pathology. There is interest in the development of improved designs which will decrease the rate of polyethylene were and mechanical failure in higher demand patients and provide increased flexibility in the primary and revision setting. The role of distal humerus hemiarthroplasty, linkable implants and components for the radial head need further investigation.

The success of elbow arthroplasty depends greatly on the surgeon’s familiarity with the anatomy and surgical approaches to the elbow joint, the proper selection and implantation of prosthetic components, and compliance with postoperative recommendations. Although elbow arthroplasty is sometimes the only option to improve pain and function in a wide range of patients, this procedure may be associated with complications which may be difficult to solve, including infection, extensor mechanism dysfunction, periprosthetic fractures, wear, loosening and osteolysis. Fortunately, revision techniques developed over the last few years allow successful treatment of some of these complications.

## Figures and Tables

**Table 1:** Main Implants Available for Replacement of the Elbow Joint

Linked	Unlinked	Linkable

Coonrad-Morrey	Capitellocondylar	Acclaim
Discovery	iBP	Latitude
GSB III	Kudo	
Norway	Norway	
Pritchard Mark II	Pritchard II (ERS)	
Pritchard-Walker	Sorbie	
	Souter-Strathclyde	

**Table 2:** Advantages and Disadvantages of Linked and Unlinked Prosthesis

	Linked	Unlinked
Advantages	Ensure joint stability May be used in the presence of ligamentous insufficiency May be used in the presence of severe bone loss Better range of motion (soft-tissue release and non-anatomic implantation)	Less constrained implants may be associated with a lower risk of wear, loosening and osteolysis Less bony-invasive, which may be beneficial if revision or resection are required Some anatomic humeral components may be used as hemiarthroplasty
Disadvantages	Increased constrained may result in increased tension to the interface and higher risk of mechanical failure secondary to wear and/or loosening More extensive canal invasion, potentially complicating revision surgery Cannot be used as hemiarthroplasty Component linking may make implantation more difficult Possible failure of the linking mechanism	Most require more accurate component positioning in order to ensure proper articular tracking It is possible to subluxate or dislocate the joint Difficult to use when there is the need to compensate for bone loss or ligamentous insufficiency Limited ability for soft-tissue release or non-anatomic implant positioning in patients with stiffness

**Table 3:** Main Indications for Elbow Arthroplasty

Chronic inflammatory arthropaties Posttraumatic osteoarthritis Acute distal humerus fractures Distal humerus Nonunions Extreme intrinsic stiffness/ankylosis Large posttraumatic bone defects Primary osteoarthritis (rare) Haemophilic arthropathy Haemophilic arthropathy Reconstruction after tumor resection
